# Rhodobacter capsulatus PG Lipopolysaccharide As a Potential Blocker of Toll-like Receptor 2 and 4 Activation

**DOI:** 10.32607/actanaturae.27555

**Published:** 2025

**Authors:** S. V. Zubova, Ya. V. Radzyukevich, N. I. Kosyakova, I. R. Prokhorenko

**Affiliations:** Institute for Biological Instrumentation, Federal Research Center Pushchino Scientific Center for Biological Research of the Russian Academy of Sciences, Pushchino, 142290 Russian Federation; Institute of Basic Biological Problems, Federal Research Center Pushchino Scientific Center for Biological Research of the Russian Academy of Sciences, Pushchino, 142290 Russian Federation; Clinical Hospital at the Pushchino Scientific Center of the Russian Academy of Sciences, Pushchino, 142290 Russian Federation

**Keywords:** lipopolysaccharides, Rhodobacter capsulatus, lipoteichoic acids, Pam3CSK4, TLR, CD14, cytokines

## Abstract

TLR2 and TLR4 play a key role in the development of an inflammation in response to a bacterial infection. We studied the effect of *Rhodobacter capsulatus *PG lipopolysaccharide (LPS) on proinflammatory cytokine synthesis activation by the TLR2 and TLR4 agonists *E. coli *LPS, *Streptococcus pyogenes *lipoteichoic acid (LTA), and Pam3CSK4 (a synthetic bacterial lipopeptide) in human whole blood cells. *Rhodobacter capsulatus* PG LPS was shown to exhibit antagonistic properties against the studied TLR4 and TLR2 agonists, blocking the synthesis of the cytokines TNF-α, IL-6, and IL-8. Possible mechanisms behind the suppressing effect of *Rhodobacter capsulatus *PG LPS are proposed. *Rhodobacter capsulatus *PG LPS can serve as a prototype for drugs against both gram-negative and gram-positive bacteria.

## INTRODUCTION

Pathogen recognition by blood cells is the most important stage of an adequate immune response to infection. TLR2 and TLR4 play a key role in inflammation thanks to their ability to identify certain pathogen-associated molecular patterns (PAMPs) [1]. These receptors also form and interconnect innate and adaptive immune responses. The study of the mechanisms of the functional responses of innate immune cells to different PAMPs is important in developing effective methods against bacterial and viral infections. TLR4 is a receptor specific to lipopolysaccharides (LPSs), which are the basic components of the gram-negative bacterial cell wall [2]. Ligand-specific recognition of TLR2 occurs via its heterodimerization with TLR1 and TLR6. Triacylated lipopeptides induce heterodimerization of TLR2 and TLR1, while TLR2 interacts with TLR6 and CD36 in response to diacylated lipopeptides [3]. Two of the three lipid chains of the triacylated ligand (in particular, Pam3CSK4) interact with TLR2, while the third chain occupies the TLR1 hydrophobic pocket [4]. Since the TLR6 molecule lacks a hydrophobic pocket, the TLR2/TLR6 heterodimer cannot recognize triacylated lipopeptides [5]. The ability of TLR2 to form a complex with either TLR1 or TLR6 opens the door for blood cells to interact with a wider range of microbial products. It also increases the production of proinflammatory cytokines and complicates the pathogenesis of sepsis.

LPS from the phototrophic bacterium *Rhodobacter capsulatus *PG exhibits low endotoxic activity and acts as an endotoxin antagonist [6]. A synthetic analogue of the lipid A of *R. capsulatus*, namely the drug E5531, is capable of blocking the immunobiological activity of LPSs and lipoteichoic acid (LTA) [7].

The aim of this work is to study the ability of *R. capsulatus *PG LPS to suppress the activation of innate immune cells by different TLR2 and TLR4 ligands.

## EXPERIMENTAL

The studies were carried out using whole blood of conditionally healthy volunteers aged 25 to 30 years. All volunteers provided a written consent to participate in the study. The study protocol complies with the World Medical Association Declaration of Helsinki (2013); it was approved by the Local Ethics Committee of the Hospital Pushchino Scientific Center of the Russian Academy of Sciences (No. 2 dated 10.04.2014). Peripheral blood was collected using vacutainers (Becton, Dickinson and Company, UK) treated with sodium heparin (17 u/ml) in the clinical setting.

**Blood cell activation by LPS, LTA, and Pam3CSK4**

To study the effect of LPS, LTA, and Pam3CSK4 on cytokine synthesis, whole blood was diluted in a RPMI 1640 medium at a ratio of 1 : 10 and incubated in various combinations with *E. coli *O55:B5 LPS (100 ng/ml),* Streptococcus pyogenes *LTA (1 000 ng/ml), synthetic lipopeptide Pam3CSK4 (300 ng/ml) (Sigma-Aldrich, USA), and *R. capsulatus *PG LPS (1 000 ng/ml) for 6 h at 37°C and 5% CO_2_. *R. capsulatus *PG LPS was obtained according to the previously described method [8]. To determine the antagonistic effect of *R. capsulatus* PG LPS on agonists, blood was preincubated with *R. capsulatus *PG LPS for 30 min. After incubation, either LPS, LTA, or Pam3CSK4 was added. Blood cells were then pelleted by centrifugation at 300* g *for 10 min. Supernatants were collected and stored at –20°C prior to cytokine assessment.

**Cytokine assessment**

The cytokine level was evaluated using TNF-α, IL-6, and IL-8 ELISA kits (Vector-Best, Russia) according to the manufacturer’s protocol. The optical density of the samples was determined at 450 nm on a Stat Fax 3200 microplate reader (Awareness Technology Inc., USA).

**Statistical analysis**

The statistical analysis and graphical presentation of the results were conducted using nonparametric statistics methods in Origin Pro 7.5 and Microsoft Office Excel 2010 (AtteStat plugin). The results are presented as median values with upper and lower quartiles (IQR). The statistical significance of differences between median values was determined using the Mann–Whitney test (*p* < 0.05).

## RESULTS

**Fig. 1:**
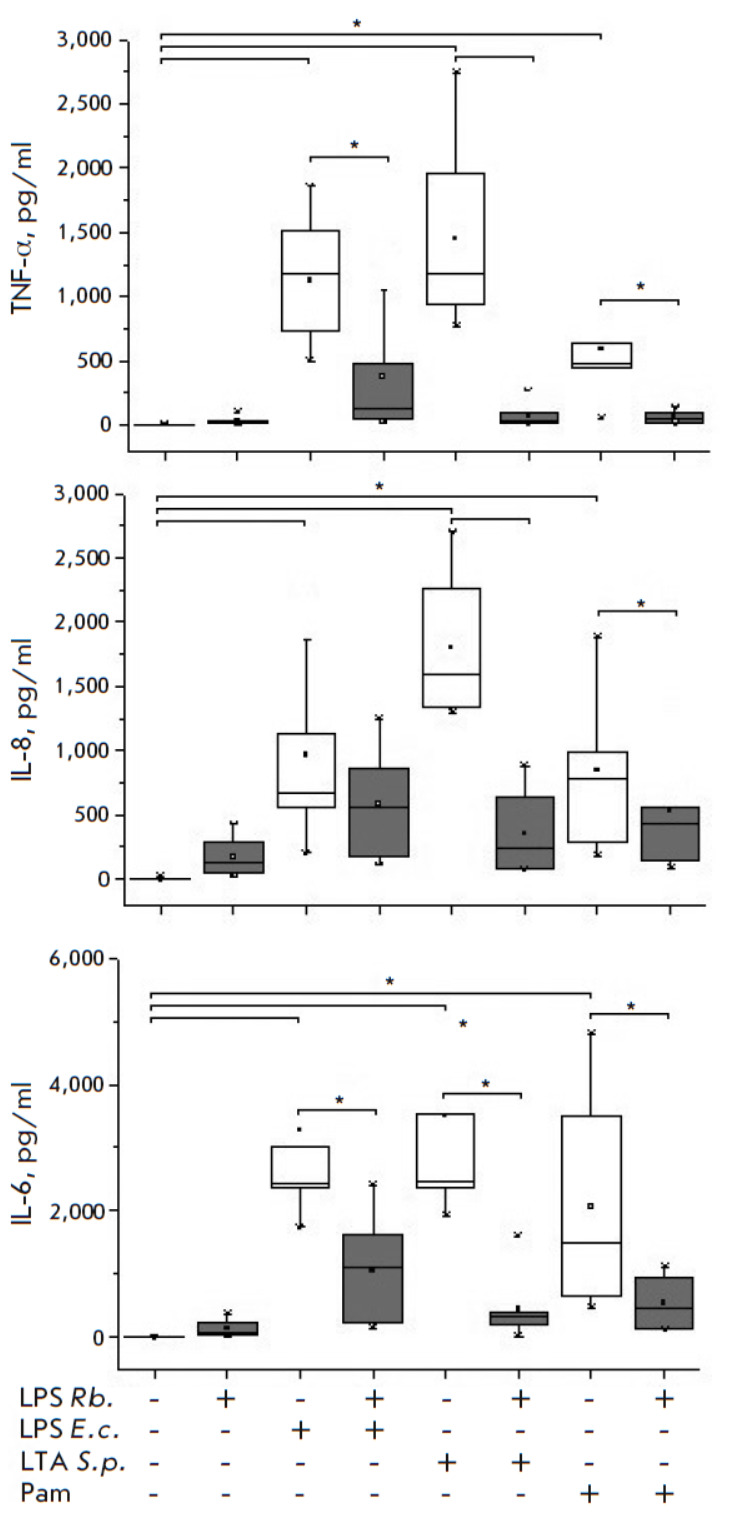
Effect of R. capsulatus PG LPS on TNF-α, IL-8, and IL-6 synthesis upon activation of whole blood cells by E. coli LPS, S. pyogenes LTA, and Pam3CSK4, n = 7. *p < 0.05

The specific receptors TLR2 and TLR4, which provide an adequate immune response to various pathogens, are the most important elements in cytokine synthesis activation. We analyzed the activation of the synthesis of the cytokines TNF-α and IL-6 and chemokine IL-8 by the following TLR2 and TLR4 ligands in human whole blood cells in a single series of experiments: *E. coli *LPS, *S. pyogenes *LTA, and Pam3CSK4. Activating ligands stimulated the production of TNF-α, IL-6, and IL-8 by blood cells at levels significantly above those in the control (*Fig. 1*).

An increase in TNF-α and IL-8 synthesis was observed in response to *S. pyogenes *LTA activation; the TNF-α and IL-8 levels were higher compared to those in the cells exposed to *E. coli *LPS and Pam3CSK4. In other words, the level of cytokines synthesized by the cells decreased in the following order: *S. pyogenes* LTA > *E. coli *LPS > Pam3CSK4. *R. capsulatus *PG LPS at a concentration exceeding that of the *E. coli* endotoxin and Pam3CSK4 by ten times and thrice, respectively, and at the same concentration as for* S. pyogenes *LTA did not stimulate TNF-α production in the cells (*Fig. 1*).

The blood levels of IL-8 and IL-6 increased insignificantly in response to *R. capsulatus *PG LPS, compared to the control; however, the levels were significantly lower than those in blood cells activated by other ligands.

The study of the ability of *R. capsulatus *PG LPS to protect cells against the action of *E. coli *LPS, *S. pyogenes* LTA, and Pam3CSK4 showed that *R. capsulatus* PG LPS suppresses TNF-α and IL-6 synthesis in the blood. The suppression of the response decreased in the same order as in the case of blood cell activation by the studied ligands: *S. pyogenes *LTA > *E. coli* LPS > Pam3CSK4. In contrast to activation by *S. pyogenes* LTA and Pam3CSK4, where a significant protective effect of *R. capsulatus *PG LPS was observed,* R. capsulatus *PG LPS did not protect blood cells from the activation of IL-8 synthesis by LPS *E. coli*.

## DISCUSSION

In this work, we studied the potential antagonistic activity of LPS from the non-pathogenic bacterium* R. capsulatus *PG not only against LPS of the gram-negative bacterium *E. coli*, which is a typical TLR4 agonist, but also against di- and triacylated lipopeptides such as LTA of the gram-positive bacterium* S. pyogenes *and the synthetic analogue of triacylated lipopeptides Pam3CSK4.

The endotoxic activity of LPSs is determined by the lipid A structure. The number of lipid chains in the structure of lipid A is the most significant factor determining LPS toxicity. It has been previously shown that E5531, a synthetic analogue of lipid A from the phototrophic bacterium *R. capsulatus *37b4, blocks the immunobiological activity of *E. coli *LPS and *Staphylococcus faecalis *LTA [7]. Unlike E5531,* R. capsulatus *PG LPS contains not only the atypical lipid A with five truncated fatty acids, including an unsaturated one, but also 3-deoxy-d-manno-octulosonic acid (KDO), an outer core, and O-antigen. The LPS inner core determines not only the LPS biological activity but also the nature of its interaction with the MD-2 protein and TLR4 [9]. For LPS recognition, TLR4 forms a dimer with the membrane protein MD-2, which binds to LPS, forming a complex capable of activating TLR4-positive cells [2]. The *R. sphaeroides* lipid was shown to occupy the entire MD-2 hydrophobic pocket, thus forming the MD-2/lipid A complex, whose stability mainly owes to the hydrophobic interaction between the lipid A tails and the amino acids of the MD-2 binding groove. Tyr102 may be responsible for the antagonist activity of lipid A due to its inverted position in the MD-2/lipid A complex [10]. MD-2 is also involved in the TLR2-mediated responses of blood cells to the wall components of gram-positive bacterial cells. MD-2 binds to TLR2; however, this binding is weaker than that to TLR4 [11].

To recognize tri- or diacylated lipopeptides, TLR2 forms receptor heterodimers with TLR1 and TLR6 [12]. Atypical LPSs of *Legionella pneumophila *and* Rhizobium spp. *induce an inflammatory response most likely via TLR2 than via TLR4 signaling [13]. In our research, we established that *R. capsulatus* PG LPS blocks the activation of cytokine synthesis in blood cells by not only TLR4 agonists, but also TLR2/6 and TLR2/1 agonists. This seems to indicate that, because of the specific composition and structure of lipid A, *R. capsulatus *PG LPS can bind not only to TLR4, but also to TLR2. Apparently, in contrast to classical agonists, the lipid A structure of *R. capsulatus* PG does not stimulate the formation of the (TLR4)2 homodimer or the complexes TLR2/TLR1 and TLR2/TLR6 required for cell activation and subsequent proinflammatory cytokine synthesis. It is possible that *R. capsulatus *PG LPS forms a TLR2/MD-2/ LPS*Rb *complex. This complex then suppresses the formation of the TLR2/6 and TLR2/1 heterocomplexes and the subsequent TLR2-mediated cell activation via LTA and Pam3CSK4, thereby increasing the production of the TNF-α, IL-6, and IL-8 cytokines.

Since *R. capsulatus *PG LPS blocks the activation of TLR4 and TLR2, we cannot exclude the mechanism of antagonistic activity proposed for *Ochrobactrum intermedium *LPS [14]. This atypical, low-toxicity LPS induces interaction of the TLR4 and TLR2 receptors and formation of the TLR4/TLR2 heterodimer upon blood cell activation. The bacteria *R. capsulatus* PG and *O. intermedium *belong to the alpha subgroup of Proteobacteria [15]. LPS from both bacteria show low endotoxic activity. The LPSs of these bacteria are comprised of lipid A containing an unsaturated fatty acid residue, an inner core, an outer core, and the O-antigen. Core saccharides are known to participate in the formation of the low-reactivity TLR2/TLR4/MD-2 complex in response to* O. intermedium *LPS [14]. It cannot be excluded that an excess of *R. capsulatus *PG LPS also induces the formation of a low-reactivity TLR4/MD-2/TLR2 complex, which blocks TNF-α, IL-6, and IL-8 production in response to *E. coli *LPS, *S. pyogenes *LTA, and Pam3CSK4.

## CONCLUSION

The results obtained here show that *R. capsulatus *PG LPS exhibits antagonistic activity against the TLR4 ligands and various TLR2 ligands, including tri- and diacylated lipopeptides. In this work, we proposed possible mechanisms behind the suppressing effect of* R. capsulatus *PG LPS on TLR2 and TLR4 activation.

*R. capsulatus *PG LPS can serve as a prototype of drugs against both gram-negative and gram-positive bacteria.

## Abbreviations

KDO: 3-deoxy-d-manno-octulosonic acid
LPS: lipopolysaccharide
LTA: lipoteichoic acid
IL: interleukin
MD-2: myeloid differentiation protein 2
Pam3CSK4: synthetic triacylated lipopeptide
PAMP: pathogen-associated molecular patterns
TLR: Toll-like receptor
TNF-α: tumor necrosis factor α

## References

[1] Mukherjee S., Karmakar S., Babu S.P.S. (2016). Braz. J. Infect. Dis..

[2] Park B.S., Song D.H., Kim H.M., Choi B.-S., Lee H., Lee J.-O. (2009). Nature.

[3] Hoebe K., Georgel P., Rutschmann S., Du X., Mudd S., Crozat K., Sovath S., Shamel L., Hartung T., Zähringer U. (2005). Nature.

[4] Triantafilou M., Gamper F.G.J., Haston R.M., Mouratis M.A., Morath S., Hartung T., Triantafilou K. (2006). J. Biol. Chem..

[5] Maeshima N., Fernandez R.C. (2013). Front. Cell. Infect. Microbiol..

[6] Prokhorenko I.R., Grachev S.V., Zubova S.V. (2010). Patent for invention RU № 2392309 of 20.06.2010..

[7] Kawata T., Bristol J.R., Rossignol D.P., Rose J.R., Kobayashi S., Yokohama H., Ishibashi A., Christ W.J., Katayama K., Yamatsu I. (1999). Br. J. Pharmacol..

[8] Makhneva Z.K., Vishnevetskaya T.A., Prokhorenko I.R. (1996). Pric. Biochim. Microbe..

[9] Prokhorenko I., Zubova S., Kabanov D., Grachev S. (2014). Crit. Care..

[10] Anwar M.F., Panneerselvam S., Shah M., Choi S. (2015). Sci. Rep..

[11] Dziarski R., Wang Q., Miyake K., Gupta D. (2001). J. Immun..

[12] Takeuchi O., Sato S., Horiuchi T., Hoshino K., Takeda K., Dong Z., Modlin R.L., Akira S. (2002). J. Immunol..

[13] Girard R., Pedron T., Uematsu S., Balloy V., Chignard M., Akira S., Chaby R. (2023). J. Cell Sci..

[14] Francisco S., Billod J.-M., Merino J., Punzon C., Gallego A., Arranz A., Martin-Santamaria S., Fresno M. (2022). Front. Immunol..

[15] Velasco J., Romero C., López-Goñi I., Leiva J., Díaz R., Moriyón I. (1998). Int. J. Syst. Bacteriol..

